# The coefficient of cyclic variation: a novel statistic to measure the magnitude of cyclic variation

**DOI:** 10.1186/1742-7622-11-15

**Published:** 2014-10-02

**Authors:** Anthony JC Fulford

**Affiliations:** 1 MRC Keneba, MRC Unit, P.O. Box 273, Banjul, The Gambia; 2 MRC International Nutrition Group, Department Public Health, London School of Hygiene & Tropical Medicine, London, UK

**Keywords:** Periodic regression, Circular data, Truncated fourier series, Coefficient of circular variation, Cosinor analysis

## Abstract

**Background:**

Periodic or cyclic data of known periodicity are frequently encountered in epidemiological and biomedical research: for instance, seasonality provides a useful experiment of nature while diurnal rhythms play an important role in endocrine secretion. There is, however, little consensus on how to analysis these data and less still on how to measure association or effect size for the often complex patterns seen.

**Results:**

A simple statistic, readily derived from Fourier regression models, provides a readily-understood measure cyclic variation in a wide variety of situations.

**Conclusion:**

The coefficient of cyclic variation or similar statistics derived from the variance of a Fourier series could provide a universal means of summarising the magnitude of periodic variation.

## Introduction

Fourier or trigonometric regression is one of the most powerful methods for the analysis of periodic data when the cycle length is known. It is a natural generalisation of the familiar cosinor regression [1]. The method fits a linear regression model in which the cyclic component, *c*(*θ*_*i*_), is represented by a truncated Fourier series, e.g.:

(1) $$\begin{array}{l} y_{i}=\beta_{0}+\beta^{T}x_{i}+c\left(\theta_{i}\right)+\epsilon_{i}\\ \;=\beta_{0}+\beta^{T}x_{i}+\sum_{j}^{P}\left[\alpha_{j}\sin\left(j\theta_{i}\right)+\gamma_{j}\cos\left(j\theta_{i}\right)\right]\\ \;+\epsilon_{i} \end{array}$$

where *θ*_*i*_ is the angle in radians corresponding to the point in the cycle at which the *i*^th^ point was measured, *α*_*i*_ and *γ*_*j*_ are the coefficients to be estimated and *P* is the number of pairs of terms in the truncate Fourier series. The *β*^*T*^*x*_*i*_ term is the linear combination of the other covariates (if any) fitted by the regression. Such models are simple to use, may be implemented with almost any statistical software and have found many and varied application (e.g. a thorough presentation by Fernandez *et al*[2]; possibly the first implementation was by Bliss [3]), although are possibly still not as widely used as they should be [4]. Their form is naturally cyclic and smooth, avoiding the unrealistic steps introduced when the period is discretised. Also, by varying the point at which the Fourier series is truncated, it is possible to determine the degree of detail fitted and avoid over-parameterising the model. Indeed, higher terms represent higher frequencies, which are often noise: by filtering them out the method automatically provides its own smoothing. By contrast, models based on discretising the cycle almost always become less realistic the more parsimonious the model. If, as is often the case, observations are reasonably uniformly distributed across the cycle, all the Fourier terms will be almost orthogonal to one another and to the intercept thus greatly simplifying model building. Furthermore, the Fourier representation is mathematically versatile allowing us under certain circumstances to deconvolve the underlying cyclic pattern when all we can observe is the cumulative effect of exposure to its influence [5].

All models of periodicity by their very nature require more than one parameter to describe them: at least one parameter each is needed in order to fit phase and amplitude. Fourier regression is no exception. Consequently the extent of periodicity is not generally represented by a single model parameter; a summary statistic needs to be derived from the fitted model. This article is concerned with the search for a suitable statistic to summarise and compare the amplitude of cyclic patterns.

## Method

A common choice of statistic to measure the magnitude of cyclic patterns is the crude difference between the maximum and minimum values of the mean. While this has its uses it also has its drawbacks: it is not always as straightforward as it may seem to locate and measure the extrema accurately or to estimate the standard error of the difference. It is also focuses on one narrow aspect of the periodic function and ignores information over much of the cycle.

Another obvious choice, at least for continuous dependent variables, would be the partial R-squared. Pewsey *et al.* review a number of exotic correlation coefficients devised for circular data [6]. These, however, essentially restrict their attention to the first pair of Fourier terms and do not take account of covariates. R-squared can be thought of as the variance of the fitted values expressed on the scale of the overall variance of the dependent variable in the sample. While this can be a logical scale to work on, it can also present a number of problems of interpretation. Often a large component of the variance is due to measurement imprecision; a scale based on the arbitrary degree of noise can have little meaning. Furthermore both precision of measurement and the underlying variance within the population will often vary between studies thus invalidating direct comparison of R-squared values. The statistical power of studies of cyclic patterns is often greatly improved by observing each individual at several different points in the cycle. In such multi-level designs there is more than one variance to consider and a difficult choice to be made as to which provides the most relevant scale on which to measure the magnitude of the periodicity.

A better choice of scale is therefore needed. We desire something familiar, universal and stable. Simply expressing the explained variance as its square root (i.e. as the standard deviation) places it on the scale of the original variable. That would be familiar and stable but not universally useful: while it might be useful when comparing the same variable measured in different studies, it is usually useless when comparing different variables even within the same study. Another approach to standardising the scale is to divide the standard deviation by mean. The coefficient of variation, frequently used to assess assay precision, is of this form, although obviously in this case it is error rather than explained variation that is being standardised. This approach is particularly appropriate when, as is often the case, proportional changes in the variable in question are important. Such variables are usually analysed in the logarithm.

Turning our attention to the variation explained by the cyclic pattern, rather than the scale on which it is measured, it is not always the case that the variance of the fitted values of the data will be appropriate. Indeed, if the data are not uniformly distributed across the cycle (had, for instance, we chosen to sample more densely where the periodic function is thought to change the most rapidly) the variance of the fitted values would not yield an unbiased estimate of the variance of an individual’s experience over the cycle. (Seasonal data associated with births may be an important exception: birth frequency is itself often seasonal and estimating seasonality of statistics associated with births, such as birth weight, might need to take that into account. An estimate of magnitude based on the variance of predicted values of the data might then be a simple solution provided the sampling frequency follows the same seasonal pattern as the birth frequency.) Instead it may often be preferable to work with the variance of the fitted function across the cycle:

(2) $${\mathrm{var}}_{\theta }\left(c\left(\theta \right)\right)=\int_{0}^{2\pi }f\left(\theta \right)c{\left(\theta \right)}^{2}\mathrm{d\theta}$$

where *f*(*θ*) is the density function. Such an approach seems reasonable and simple but is not widely used in regression analysis probably because the underlying distribution density of the predictor variables is not generally known. However, with cyclic data the relevant underlying distribution (e.g. when considering an individual’s experience over the full cycle) is usually uniform, *f*(*θ*) =1/2π, and easy to work with. Under this assumption the variance of a Fourier series over a full cycle turns out to be very simply half the sum of the squares of the coefficients. Thus, for the parameters in model (1):

(3) $${\mathrm{var}}_{\theta }\left(c\left(\theta \right)\right)=\frac{1}{2}\sum_{j}^{P}\left[\alpha_{j}^{2}+\gamma_{j}^{2}\right]$$

When the outcome variable is measured on a common, recognisable scale (e.g. an anthropometric z-score) this statistic serves as an adequate measure of the amplitude of the periodicity. In other cases it would be useful to rescale the variance. This suggests a statistic analogous to the coefficient of variation, which I will call the Coefficient of Cyclic Variation (*ccv*): sd(*c*(*θ*))/mean. Provided the mean of *x*_*i*_ in the Fourier regression model, (1), is zero (i.e. the covariates are centred) and the data are a reasonable representation of the population then the mean will be given by *β*_*0*_:

(4) $$\mathrm{ccv}=\sqrt{\frac{1}{2}\sum_{j}^{P}\left[\alpha_{j}^{2}+\gamma_{j}^{2}\right]}/\beta_{0}$$

The *ccv* is thus a simple function of the parameters in the Fourier regression model. Its standard error and confidence intervals may be readily calculated either using the delta method or the bootstrap (Stata’s *nlcom* command, for instance, can be used to estimate the statistic and its confidence interval based on the delta method – StataCorp, College Station, TX).

As mentioned above, scaling by the mean is most appropriate when proportional changes in the variable are important. In these cases the data are often analysed in the logarithm, i.e. *y*_*i*_ is replaced by log(*y*_*i*_) in equation (1). In that case, provided sd is small relative to the mean, the *ccv* for the original untransformed variable is given approximately by:

(5) $$\mathrm{ccv}\approx \sqrt{\frac{1}{2}\sum_{j}^{P}\left[\alpha_{j}^{2}+\gamma_{j}^{2}\right]}$$

### Two examples

**Figure 1:**
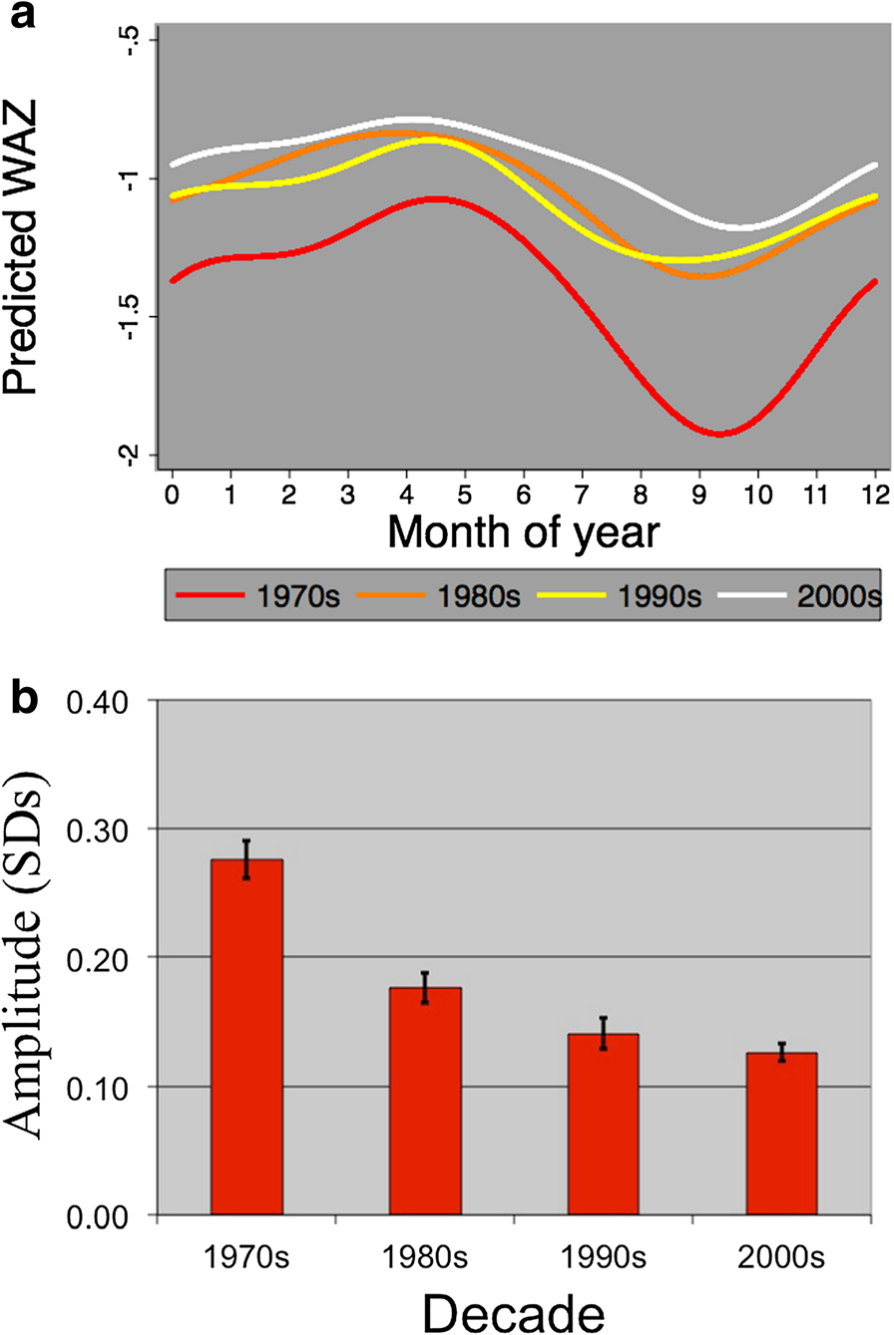
**Declining seasonality of weight-for-age Z-scores (WAZ). (a)** Seasonal patterns for each decade. **(b)** Estimated amplitude vs decade. Predicted WAZ for under-2 year old children in Keneba, Kiang West, The Gambia. These data were derived from approximately 10,000 observations on 800 individuals (boys and girls combined; their pattern were very similar) each decade. The first three pairs of Fourier terms were used to fit the curves for each decade. The z-scores were based on the WHO 2006 standard tables.

**Figure 2:**
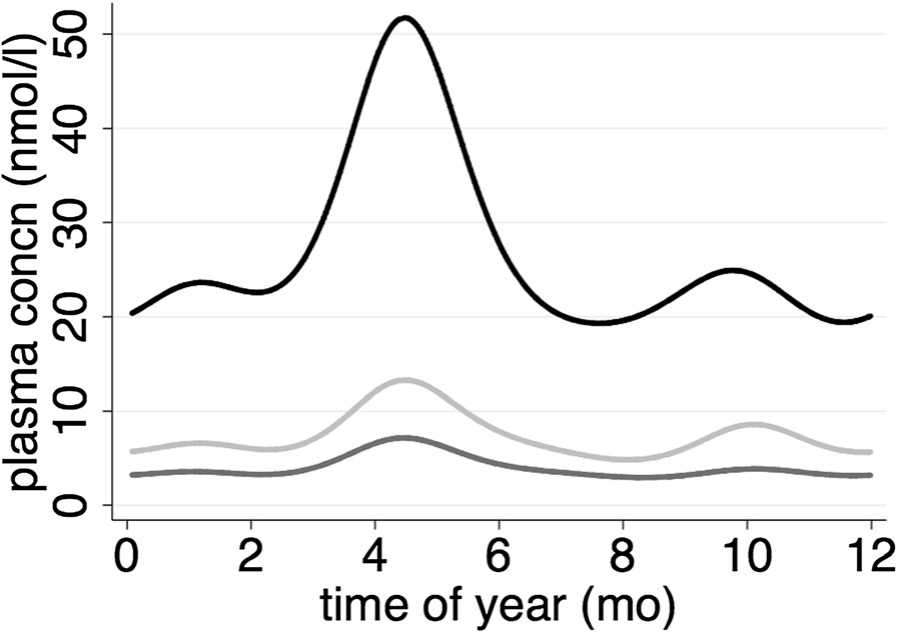
**Seasonal patterns of vitamin B6 biomarker levels in plasma of Gambian women.** Pyridoxal = light grey; pyridoxic acid = dark grey; pyridoxal phosphate = black. The data were derived from 315 samples on 52 women observed across the year. Curves show the plasma concentration (mg/dl) estimated using generalised least squares random effects fitting the first four Fourier terms.

1. The use of the unscaled variance is illustrated by the example of the change in seasonality of weight-for-age z-scores among Gambian children in recent decades. Figure 1a shows how not only has wasting in these children reduced since the 1970s but the children also appear to be less susceptible to seasonal changes (probably buffered by the “remittance economy”). This is confirmed by the plot of the amplitude of seasonality shown in Figure 1b.

2. This example employing the *ccv* is shown in Figure 2. Here the seasonal patterns of plasma pyridoxal, pyridoxal phosphate and pyridoxic acid derived from 315 observations of 52 Gambian women (data courtesy of Paula Dominguez-Salas). The logarithms of the assay values were fitted by random effects GLS regression using the first four Fourier terms and controlling for age; the fitted values were anti-logged to yield the plotted curves. All three biomarkers appear to follow the same seasonal influences peaking in May but are they affected equally? Although a thorough analysis would make allowance for the fact that these biomarkers were measured in the same women, from the *ccv*s and their 95% confidence intervals tabulated in Table 1 it is clear that the seasonal patterns are of very similar magnitude. Note also the consistency between the two methods used to estimate the confidence intervals.

**Table 1:** The seasonal variation in vitamin B6 biomarkers among Gambia women

**Biomarker**	***ccv***	**CI 95%**	**CI 95%**
		**(delta method)**	(bootstrap*)
Pyridoxal	25.3%	(21.9%, 28.6%)	(21.5%, 28.9%)
Pyridoxic acid	27.3%	(23.3%, 31.4%)	(22.6%, 32.0%)
Pyridoxal phosphate	29.0%	(25.0%, 33.0%)	(25.0%, 33.1%)

*Employing 50 re-samplings of the data.

## Conclusion

There are numerous ways to exploit the simple (but apparently little known) formula for the variance of a Fourier series given in equation (3). I suggest that in many, probably the great majority, of cases when seasonal or diurnal patterns are investigated in epidemiological studies, the *ccv* or related statistic will provide a simple and useful measure of the size of periodic variation. Its simplicity has the potential for this approach to provide the universally recognised standard statistics to be reported in such studies.

## Competing interests

The author declares that he has no competing interests.

## References

[B1] Nelson W Tong YL Lee JK Halberg F Methods for cosinor-rhythmometry Chronobiologia 1979 6 305 323 548245

[B2] Fernández JR Hermida RC Mojón A Chronobiological analysis techniques. Application to blood pressure Phil Trans R Soc A 2009 367 431 445 10.1098/rsta.2008.0231 18940774

[B3] Bliss CI Periodic regression in biology and climatology Bull Conn Agric Exp Station New Haven 1958 615 1 55

[B4] Cox NJ Speaking stata: in praise of trigonometric predictors Stata J 2006 6 4 561 579

[B5] Fulford AJC Rayco-Solon P Prentice AM Statistical modelling of the seasonality of preterm delivery and intrauterine growth restriction in rural Gambia Paediatr Perinat Epidemiol 2006 20 3 251 259 10.1111/j.1365-3016.2006.00714.x 16629700

[B6] Pewsey A Neuhäuser M Ruxton GD Correlation and Regression Circular Statistics in R 2013 Oxford: Oxford University Press 149 170

